# In search of finalizing and validating digital learning tools supporting all in acquiring full literacy

**DOI:** 10.3389/fpsyg.2023.1142559

**Published:** 2023-08-04

**Authors:** Heikki Lyytinen, Natalia Louleli

**Affiliations:** ^1^Department of Psychology, University of Jyväskylä, Jyväskylä, Finland; ^2^Basque Center on Cognition, Brain and Language BCBL, San Sebastian, Spain

**Keywords:** reading, full literacy, ComprehensionGame, GraphoGame, association learning

## Abstract

Unlike many believe, accurate and fluent basic reading skill (ie. to decode text) is not enough for learning knowledge via reading. More than 10 years ago a digital learning game supporting the first step towards full literacy, i.e., GraphoGame (GG) was developed by the first author with his colleagues in the University of Jyväskylä, Finland. It trains the acquisition of basic reading skills, i.e., learning to sound out written language. Nowadays, when almost everyone in the world has an opportunity to use this GG, it is time to start supporting the acquisition of full literacy (FL). FL is necessary for efficient learning in school, where reading the schoolbooks successfully is essential. The present plan aims to help globally almost all who read whatever orthography to start from the earliest possible grade during which children have learned the mastery of the basic reading skill to immediately continue taking the next step to reach FL. Unlike common beliefs, support of FL is mostly needed among those who read transparent orthographies (reading by the majority of readers of alphabetic writings) which are easier to sound out due to consistency between spoken and written units at grapheme-phoneme level. This makes readers able to sound any written item which is pronounceable with only a little help of knowing what it means. Therefore, children tend to become inclined to not pay enough attention to the meaning but concentrate on decoding the text letter-by-letter. They had to learn from the beginning to approach the goal of reading, mediation of the meaning of the text. Readers of nontransparent English need to attend morphology for correct sounding. The continuing fall of OECD’s Program for International Student Assessment (PISA) results, e.g., in Finland reveals that especially boys are not any more interested in reading outside school which would be natural way to reach the main goal of reading, FL. What could be a better way to help boys towards FL than motivating them to play computer games which requires reading comprehension. The new digital ComprehensionGame designed by the first author motivates pupils to read in effective way by concurrently elevating their school achievements by connecting the training to daily reading lessons. This article describes our efforts to elaborate and validate this new digital tool by starting from populations of learners who need it most in Africa and in Finland.

## 1. Introduction

The objective of this articles is to review our efforts to support reading skills from the beginning of reading acquisition to full literacy. Reviewed here is a large number of our studies from which we pick only the most important information to help reader to follow how we try to approach our objective: help learners to acquire full literacy (FL). We summarize our theoretical and empirical observations related to supporting the ways children are supported in practical terms. This means description of the ways how our two digital game-like learning environments support first the basic reading skill and then how our latest game also helps learners to reach the main goal of reading, full literacy (FL). All the support is built to be open to be used globally via net by almost everyone in need, because the training works in cheap devices which require only occasional connection to internet.

The common features of these two digital learning environments are our attempts to make learners: (1) focus on the key features which have to be learned, (2) engaged to repeat until the needed learning has happened, and (3) getting immediate feedback (with as little as possible negative feedback). We emphasize the need of making the games empirically proven to be effective before being ready for wider use. The new learning game illustrated here first time is ComprehensionGame. It is designed to help in reaching the FL. It is language independent and open to support learners of any age after the user has acquired mastery of the basic reading (decoding) skill.

Two steps of learning are needed for full literacy as documented long time ago by Hoover and Gough (1990) in their “Simple view of reading” paper but which has not got the attention it deserves. One has to reach the goal of reading which is comprehending the message the text is mediating. This is not, however, possible without first learning the basic reading skills which is the most demanding to those who face reading difficulties or dyslexia. We studied related difficulties in detail in our Jyväskylä Longitudinal study of Dyslexia (JLD, see, e.g., Lyytinen et al., 2006; Lyytinen, 2014; Eklund et al., 2015). Later we observed that it is caused by genetic factors affecting the brain in such a way that differentiation of sounds is compromised (Leppänen et al., 2010; Lohvansuu et al., 2021) in a way which can be corrected only by heavy drilling (Niemelä et al., 2020), which seems to be possible using our GraphoLearn technology (GL) which we developed for training the basic reading skills. It was used to build GraphoGame (GG; development of which was supported the work of e.g., Kujala et al., 2010a,b), now available with this name under the label we used when we published its development and validation research (for review, see Lyytinen et al., 2021). Its Finnish version is called Ekapeli (Lyytinen et al., 2009) which has been shown to help all in need successfully [see Saine et al. (2010, 2011, 2013) for a detailed description of the Ekapeli and its efficiency]. Its important feature is that it is engaging and providing positive feedback (Ronimus et al., 2014) thus helping also children who face severe difficulties in learning the basic reading skill (Ronimus et al., 2020) if given preventively. How it should be used applying the results of the JLD-study is described (e.g., in Lyytinen et al., 2015). It works if used before facing the problems which may start avoidance behavior if it has had time to develop due to experience of failures especially if teacher has asked the child to read aloud when other classmates are observing the situation. Only part of children tolerates such an experience without starting to avoid practicing reading (Aro et al., 2009) and thus but not if started to late (Ronimus et al., 2023). Now the GraphoGame used outside Finland is out of our hands because the university could not maintain the worldwide servers. It is nowadays available from www.graphogame.com.

Depending on the orthography, learning and training the basic reading skill differ substantially. In the fully transparent writings, which behave consistently at the grapheme-phoneme level, it is very easy to learn if instructed optimally, which means first of all that children at risk of failing had to start using it preventively but not before the time children had to start learning to read. Used this way our GraphoLearn technology has been shown to train practically anyone to acquire the basic decoding skills. Its results have been documented also via brain-related observations (Brem et al., 2010). The only difference between learners is the time needed depending on many reasons which can compromise one’s acquisition of the basic reading skill. The compromising factors vary a lot. These the most common include (1) the quality of teaching where a poor traditional teaching as the only compromising factor makes one to fail but learning using the game fast and (2) learners’ readiness to learn which may be affected by genes and thus atypical brain activity called dyslexia. Even children with severe dyslexia can be helped if the game is used optimally. As shown in the mentioned review the GraphoGame has been helping successfully in tens of countries in languages for which it has been made to work which include English, German, Spanish and some African languages. Reaching such a success has required that the first author and his colleagues in the University of Jyväskylä have been helped by locals to implement the language’s sounds and the letters/graphemes representing these – most mentioned in the review. The most important colleagues have been Ulla Richardson from our university and Usha Goswami from Cambridge University.

A typical Finnish child who has not learned to read before school can acquire the decoding skills to sound out whatever pronounceable written item with less than two hour’s training using short, repeated training sessions of the Ekapeli during first weeks of the first semester of school. Children with dyslexia may need many hours but the less the more optimally the game is used. GG/Ekapeli may not suffice to overcome the problems if not used preventively. Optimal timing is to start the use immediately when one enters school and play it in successive days 2–3 times per day for no more than 20 min at time until the basic reading skill is acquired. E.g., in Finland practically all are playing Ekapeli. Finnish children reach accurate decoding skill not later than during the first months of school.

In Africa, where reading instruction is compromised, e.g., in Zambia, also teachers had to use it for being able to let her/his pupils to benefit from the use of GG optimally as we will show here. The main emphasis in this article is, however, in the description of our latest learning game, ComprehensionGame, which is designed to support readiness to learn knowledge via reading. It is recommended to be used in the context of learning lessons from the schoolbooks starting from the time children master the basic reading skill. Its empirical basis has been documented e.g., by Louleli et al. (2022) and Lyytinen and Louleli (2023).

### 1.1. How the GraphoLearn technology helps learners to acquire the basic reading skill

The first step towards full literacy training the basic reading skill happens using the GraphoGame. It instructs children to connect spoken items to written items emphasizing the learnable connections, i.e., those which are as often as possible true. In this it follows the theory described by the first author, e.g., in the mentioned review that learning to read any language is based on association learning, connection building between spoken and written units. Identifying such connections in transparent writing is easy. Learners have to get training to connect each letter or 2–3 letters’ grapheme with the sound it represents.

**Figure 1:**
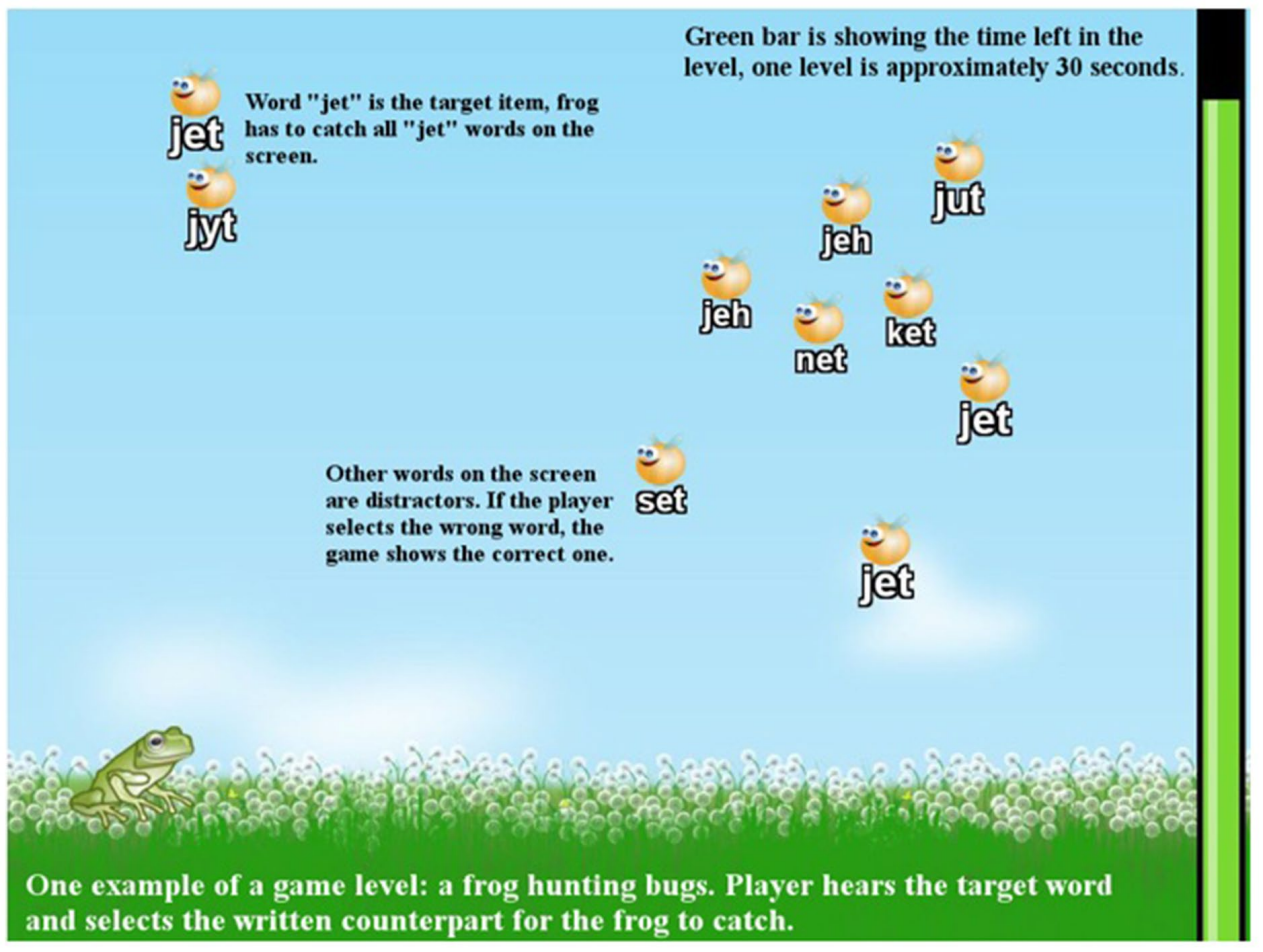
In the English GraphoGame the choosing of corresponding – here larger – written units to represent the one the learner hears from the headphone is occurring in this type of seen.

In nontransparent writing systems, such as English, this is less easy. By computing we can observe the nature of the problems related to the needed consistency of the connections. At the level of single letters, it is very low in English (see Table 1). As shown, practically none of the letters in the English writing represents the same sound everywhere. Thus, one has to choose larger units, such as rimes for this connection building (Ziegler and Goswami, 2005). For example, an easy-to understand larger unit is the letter sequence “ing”, which is sounded out the same way in all contexts of English writing. Implementing the game by using such items in the game as shown in Figure 1, we can understand how the connection building can be trained using the basic from of association learning. The learner hears a sound (spoken item) from headphones and at the same time s/he sees a number of written items on the display where one has to be chosen in such a way that it is the one representing the heard spoken item. Proceeding from easy to more difficult connections, the learner slowly becomes able to store the connections and also to assemble smaller items together to approach via syllables to the word level. This procedure has been shown to train the basic reading skill of English faster than any other instruction among typical learners (Kyle et al., 2013) as well as among children who have difficulties in learning to read (Ahmed et al., 2020).

**Table 1:** An example of the statistical approach to illustrate the problems associated with consistency (or the paucity of it) in English.

Letter	(%) of different / all words (exemplary word)			
i	62.3	24,076	3,471,217	I(in)
	19.4	4,386	1,083,446	aI(i)
	5.1	2,519	283,459	(social)
l	95.4	22,272	2,934,160	l(all)
d	94.4	14,990	2,844,232	d(and)
m	100.0	11,176	1,817,206	m(from)
b	99.0	7,726	1,169,525	b(be)

A minimum set of single letter-sounds selected to a version of the game – list of their sounds present in >5% of the occurrence of the letter in English text (Cedex databasis, among 17 million words).

It is interesting that when the theory emphasizing the central effects of phonological skills was presented one of its proponents (Shankweiler and Fowler, 2004) wanted to inform teachers how it had to be understood his and Ann Fowlers article had a very closely associated interpretation (to my connection building idea) in mind but it looks that none read it carefully enough for being able to follow it. My theory is based on the basic theory of learning (association learning) but experts of reading research were not connected to experts of learning research at that time but Donald and Ann spontaneously ended up understanding that.

Above we learned that the acquisition of the basic reading skill can be effectively supported by applying the basic learning theory, the association learning known for decades. This almost self-evident fact has not been explicitly presented in reading research because it has been affected by the trends of learning research which has emphasized for years into the cognitive aspects of learning common in most descriptions given in reading literature concerning almost exclusively the way learning to read English.

As it can be understood from the afore mentioned, the learning load of acquiring the basic reading skill varies between orthographies. The closest simplified metrics about the load is the number of consistent (or close to be consistent) connections between spoken and written language. In fully transparent writings, it is always much less than one hundred, in English several hundreds and in, e.g., Chinese writings thousands. In the last, the GraphoGame learning could be made working by connecting the orthographic units to spoken units. Only the Pinying versions of GraphoGame exist and they have been shown to instruct the sounds and their connections to written Chinese very fast (Li et al., 2017).

**Figure 2:**
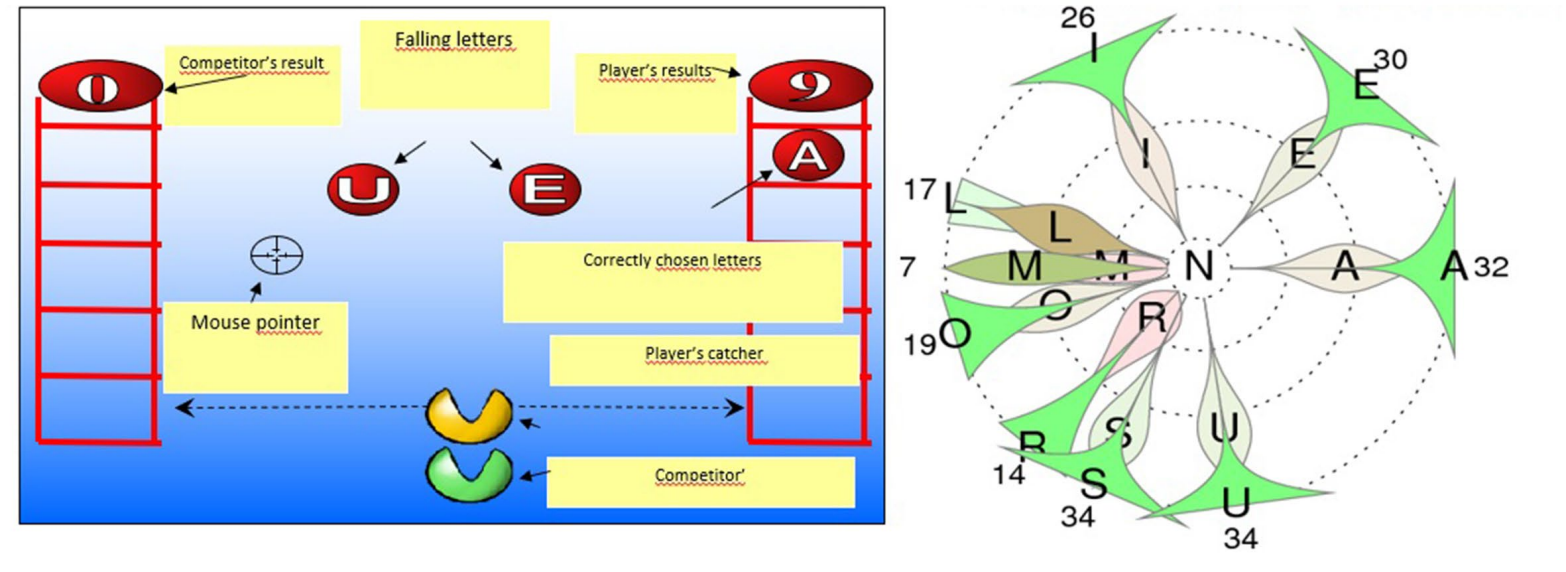
Graphogame – an example of the classical version of the Finnish game helping in learning the connections between spoken and written language from letters to words. Description: In the game (left) the learner is choosing (in its classical version) from the falling balls the corresponding letter of the one s/he hears from headphones. The illustration (right) shows an example of how results can be followed. Here we follow how /N/ sound (in the centre) which learner has heard in the game more than 100 trials at the moment this picture is printed from the game logs has made him/her to choose incorrect alternative letters (shown with the number of times these have occurred with the correct N-letter). The red distributions reveal that the learner has had difficulties in not to choose R and M during the first fourth of such trials, but became able to learn during the last fourth (with green distribution) that e.g. R does ot represent the /N/ sound. For this learner acquiring that /N/ sound is not represented by M-letter has been a real challenge as shown by the red and darker green distributions, which reveal the most of the choices during the first and second fourths of trails (respectively) have ended up to this mistake. The learner has failed to learn to identify the correspondence of the /N/ sound during the whole session in trials where M has occurred (7 times) as an alternative. On the other hand s/he has not chosen. e.g. S to represent the /N/ sound any more during the last fourth of the trails (no misidentifications during the 9 last of the 34 trials with S as an alternative). For more details, see Lyytinen et al. (2009) and for documentation of the efficiency of the game in supporting learning among at risk children, see e.g. Saine et al. (2011) from which it is modified.

For imagining how the GraphoGame works the reader can skim the Figure 2 which shows the classical version of the game as implemented for Finnish learners. The right-side figure shows how the results can be followed at any time.

### 1.2. How children in sub-Saharan Africa can learn the basic reading skill?

In the Sub-Saharan Africa, the situation associated with learning the basic reading skill is complicated for example because children had to learn to read first the language which they speak which helps them most easily to understand the alphabetic principle. In most of these countries, today’s teachers were instructed to read English during their first school years. Although it was not made with very good results all learned the way how learning to read English was attempted by staring from eg, using songs of the names give to the letters.

Almost only 20 years ago, the focus of reading instruction was moved to local languages in Sub-Saharan Africa. This was made as widely as it should be done (see, e.g., Lyytinen et al., 2019a,b) by choosing the most commonly spoken languages children speak in the area of their school. For example, in Zambia the move has not led to very good results because teachers are still relying on the instruction which they experienced during their school years for learning to read English. Thus, a few years ago when we studied the results of reading instruction for the acquisition of the basic reading skill using Early Grade Reading Assessment tool (EGRA), we found very poor learning results (Sampa et al., 2018). Most scores were zeroes for almost all the basic measures of EGRA.

**Figure 3:**
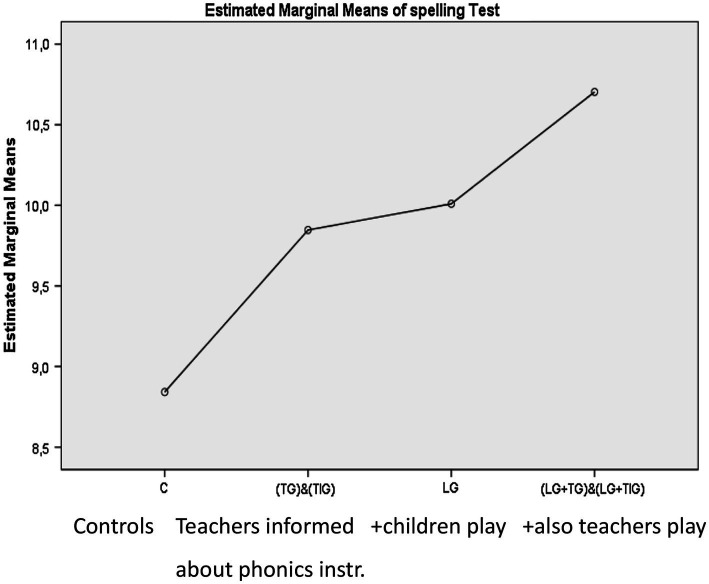
How Graphogame helps in Zambia. Modified from Jere-Folotiya et al. (2014).

This motivated us to try the GraphoGame for helping Zambian leaners by starting from the validation research of its efficiency. For that we opened a support center called Centre for the Promotion of Literacy in Sub-Saharan Africa (CAPOLSA) which is working still now in the context of the University of Zambia (Lyytinen et al., 2019a,b). And in fact, the results of its first local worker (Jere-Folotiya et al., 2014; Nshimbi et al., 2020) and (Ojanen et al., 2013, 2015 for review) reveal that it is realistic to reach good results on the condition that both teachers and learners use it (see Figure 3). Still today it is difficult to motivate teachers to do that, but those who agree, can get good results from the resulting instruction based mainly on the GG.

A second dissertation which is under its finalization phase in Zambia made by Jonathan Munachaka reveals that the typical portion of children who have problems due to biological reasons, i.e., dyslexia, is there the same it is in the world (<5%). This can be observed using GG as dynamic assessment (DA) tool. It shows how many repetitions learner needs for store a few connections between spoken and written items. Those who have no problems in connecting sounds to their written forms learn how to read via GG not only their local language but also are able to train themselves successfully to read English. The dissertation reveals that the small (5%) portion who shows poor readiness to learn these connections in DA during their early school years, fail to learn to read before the end of sixth grade any language they are instructed to acquire in Zambia.

### 1.3. Learning the final step towards full literacy

Learning the basic learning skill is not enough for learning knowledge by reading. Therefore, we have most recently concentrated on R&D related to training learners to reach the goal of reading, i.e., full literacy which is best defined via the procedure used for OECD’s PISA.

Possibilities to learn knowledge using local African languages is highly compromised due to several reasons. The first is the insufficient instruction to learn the basic reading skill. A next is the very poor availability of appropriate knowledge books, or also the lack of needed skills for reaching the goal of reading (FL). The natural way to acquire FL is reading a lot, but this is not possible in Africa due to lack of interesting reading material (leisure reading) which could be used outside school.

These problems are now open to be solved via digital technology and Internet. Digital tools can be made available to all soon for training the reading skills to reach FL and opportunity to access Internet is readily available in most locations in Africa and becomes available to all soon to open doors to knowledge. Therefore, our attention has now been focused on training Zambian people to reach FL using our new ComprehensionGame (see comprehensiongame.com).

### 1.4. What we know about reading comprehension and ways to support it: a selective summary of existing knowledge

Reading is a non-innate readiness starting from the decoding of written to spoken language. Its basic form reading accuracy and fluency skills belong to the basic reading skills which can be supported using the GraphoGame. Only after acquiring the basic reading skills one can start taking the final step for reaching full literacy. The failure experiences while taking the first step tend to make children less interested in leisure reading which is a necessity for learning to comprehend text in natural way. When one has acquired FL s/he had to comprehend written language as well or better than he/she does comprehend spoken language which is the goal of training the second step towards full literacy.

Nowadays, there are many theoretical frameworks that have been tried to disentangle the reading comprehension development, such as the lexical legacy hypothesis (Nation, 2017), the reading systems framework (Perfetti and Stafura, 2014), the direct and mediational inference model (Cromley and Azevedo, 2007), and the construction–integration model (Kintsch, 1988).

Many studies have attempted to entrain poor readers’ comprehension skills via intervention studies. Many intervention practices suggested for poor readers have derived from observing, questioning, and asking good and poor readers to “think aloud” while they read (Dole et al., 1991; Jiménez et al., 1996). Other studies have shown that explicit strategy instruction generates strong effects for comprehension for students with learning difficulties (Gersten et al., 2001; Biancarosa and Snow, 2004), that effective comprehension instruction in the elementary grades teaches students to summarize, use graphic organizers, generate and answer questions, and monitor their comprehension (Mastropieri et al., 1996). As far as the readers with typical reading skills are concerned, the most common interventions in the literature are those that try to teach readers some strategies to improve their comprehension skills, such as comprehension monitoring, inference making, cooperative learning, question generating and answering, identifying main idea, summarizing, predicting, and recognizing the structure (Gersten et al., 2001; McKeown et al., 2009; meta-analysis: Jamshidifarsani et al., 2019). For example, aurally presented material were very commonly used, as they suggest that comprehension skills are not specific to the medium in which narratives are presented, but are similar across different media (Kendeou et al., 2005). For example, in their early study of Reciprocal Teaching, Palinscar and Brown (1984) successfully improved reading comprehension skills of pre-readers and children with reading difficulties via aurally presented texts. Similarly, Fuchs and colleagues (Fuchs et al., 1997) in the development of Peer Assisted Learning Strategies (PALS) also used aurally presented material for the development reading comprehension skills in students with special needs. Another intervention study by Capodieci et al. (2021) also investigated the effects of a training program focused on the comprehension abilities of first grade children by training the auditory or written comprehension in comparison with a control group, which did not receive any training (Capodieci et al., 2021). Their results demonstrated that both the training programs were benefited from the listening and reading comprehension as compared with the control group, and that the that the achieved benefits on reading comprehension were maintained within a five-month period (Capodieci et al., 2021).

Moreover, a more recent intervention, which trained the comprehension skills of school children with reading disabilities, used the self-regulated strategy development (SRSD); even though the results showed that this strategy had generally effective results, the limited number of studies did not allow this strategy to be further used in a bigger sample of school children (meta-analysis: Sanders et al., 2019). Another intervention study investigated the effects of a motivational training in the enhancement of reading comprehension skills (McBreen and Savage, 2022). Specifically, this intervention managed to assess two training groups twice (pre and post training): the first one was offered a cognitive training, including phonological awareness, listening and reading comprehension, etc., while the second group was offered a cognitive plus motivational training targeting on students’ emotions, beliefs, and self-efficacy (McBreen and Savage, 2022). Their results showed that a set of cognitive reading intervention together with the proposed motivational program can improve the reading performance of students at risk for reading difficulties (McBreen and Savage, 2022).

Many research studies focused on interventions targeting reading comprehension skills, the ultimate goal of reading. Various skills are known to be involved in proficient reading comprehension, so an impairment in reading comprehension deficit has been probably resulted from a deficit manifested to one or multiple of these skills, such as: lexical processes (including phonological skills, semantic skills, and visual word recognition), working memory, cognitive inhibition, attention allocation, inference making, comprehension monitoring, and general knowledge (Nation, 2005; Kendeou et al., 2014). Therefore, each individual with reading comprehension difficulties can have a different underlying problem, resulting in a different reading profile (Cain and Oakhill, 2006). Here, we, however, focus our attention on comprehension skills which had be trained to be equal or better by reading than by understanding spoken language. Thus, our focus is not including lexical skills, but on how the available lexicon can be benefited also for comprehending written knowledge based on learners existing spoken lexicon.

The afore-mentioned approaches illustrate theoretical and empirical observations of skills related to reading comprehension or developmental pathways or conditions that reading comprehension seems to follow. We have been unable to find any published application of training the reading comprehension-related skills which would be open to be given to help everyone independently of language, age or initial skill. Thus, our plan to offer a possibility to train all in need in the world using ComprehensionGame has not found any competitor yet. ComprehensionGame (CG) covers of replaces the need of many of the key features mentioned to be used for training the comprehension of written material as we try to inform below.

The gap between the previous studies and our study is that the former ones have used aurally presented materials, which have not been open to be implemented in forms which can boost children’s comprehension skills in whatever language and in all places where learners can connect their devices to internet. CG also makes the learning possible by emphasizing on the engagement especially due to its gamified versions. Specifically, the CG will increase each child’s interest in training him/herself with a game-like environment in-order to become a more and more active reader. This activity is trained to focus on searching answers which the title of the text guides her/him to find, so that at the end s/he would master the key knowledge which, e.g., the daily lessons from the schoolbooks are including and offering with the support of CG. Possibly the most important single feature of the CG is that it guides the learner to identify the key contents from the text. Only a limited number of sentences can be kept at time in the working memory which is needed for integrating the knowledge for following the story line efficiently. This is why the key contents can be identified efficiently and compressed to a form which prevents the overflowing of the working memory.

The logical features most literature of reading comprehension is listing are the importance of the background knowledge and readers’ active search for meaning when reading text. The last is learned naturally via leisure reading while the background information can be “controlled” well enough in training reading comprehension by proceeding according to the school program which is elevated step by step following the curriculum. Especially the first requirement is seldom met in Africa where leisure reading is not possible to many learners in Sub-Saharan Africa before a sufficient master of reading of a second language has been acquired. Therefore, especially in those poor countries the kind of CG training of reading comprehension is needed. And this in fact has been the starting point also of the development of the ComprehensionGame for African users.

Interestingly, most of the afore-mentioned intervention studies are mainly focused on the children with reading difficulties, but what about the less privileged African children? No studies have mentioned so far the CG’ ambitious goal to develop a reading tool in order to help appropriately and efficiently each learner of any age, country, culture and educational level. Thus, the training tools had to be able to also help, e.g., African illiterates by providing the basic reading skill and then knowledge according to each individual’s needs, i.e., accepting her/him to have a good opportunity to choose content s/he very much wanted to read at the top of the obligatory texts connected to lessons from the schoolbooks.

Another very important aspect affecting the successful acquisition of reading comprehension skills is the role of the teacher. Usually, the most popular strategy among teachers to teach/guide their students to reading comprehension is the guided comprehension strategy. Guided comprehension is a context in which students learn and use comprehension strategies in a variety of settings in which multiple levels and types of text are used. The strategies taught in Guided comprehension include the following aspects: pre-viewing, self-questioning, making connections, visualizing, monitoring, and summarizing, evaluating. This guided instruction strategy for comprehension is theoretically each teacher’s duty to follow in-order to help each student with reading comprehension, followed by a gradual release of responsibility to each student later. These duties belonging to teachers have been implemented in the ComprehensionGame development system using a separate content creation tool called Creator. Using that also less educated teachers can learn to elevate their teaching skills because it guides her/him to consider what is the teachers duty in the process. This all is connected especially to the key information belonging to the learning content specified in the curriculum, which is readily implemented in the game. This available content instructs teachers to continue the implementation of one’s own content or content of interest to learner, when needed.

The effectiveness of the teaching method has to consider the differences in the reading levels of students as well as the individual needs of each student. The CG is able to motivate teachers to implement the key contents from the schoolbooks via tablets in a form which is meant to be individually helpful for each student depending on her/his readiness to follow text. Also, the application of dynamic assessment helps in making the training adapted to needs of any individual.

Because engagement in training one’s skills can be defined as “the mother” of all learning this application is made to optimize it by providing immediate feedback about learners performance (to both teachers and learners themselves) by using a “reward-point” system which can be used in many ways, e.g., by letting one to earn time for playing games s/he prefers playing which are not meant to complement CG but work as rewards from concentrating also in the use of the CG. Furthermore, the CG environment is able to elevate the engagement by making competition possible between the learners in such a way that everyone competes within her/himself and the proceedings are compared between individuals. This way, it will be possible to get rid of the traditional school exams by showing during learning the lessons learned, thus elevating the equality between individuals because at the end all the learners will be able to reach the same goal (although most likely they would need different times for reaching the goal).

Possibly the most motivating aspect of the CG is that it can help everyone to learn the lessons well enough in shorter time. This is because the game focuses the learners to concentrate on the key contents of the lesson. It also saves learners’ and teachers’ time in helping learning/instructing the knowledge contained in the curriculum.

Overall, many previous intervention studies have tried to train the comprehension skills of children (with and/or without reading difficulties) by using different strategies and means and often their results have been positive. However, the CG intervention tool provides more benefits than the more traditional interventions, with the most important being its global availability to prepare children to learn the whatever knowledge they have in their schoolbooks via reading in every language and age. The way the key information is introduced can be given to be used using the implementation of content which the most experienced, and thus effective and skilled teachers have done (if they accept that as we believe). And a further acute need is also fulfilled because the game helps children to approach any written material with critics – a very important readiness needed more and more today. The CG will be able to extend the learning of illiterate learners in combination with the GraphoGame to achieve full literacy, e.g., in Africa, as we have shown. And a further mentionable benefit is that it motivates teachers to be better prepared to instruct learners when it requires them to train themselves to identification and implementation of the most important content of any lesson to her/his pupils to learn.

### 1.5. The starting principles we follow when preparing ComprehensionGame

Below we try to describe in more detail the working of the CG we first list the goals that it tries to approach, and the means used to reach these goals – understanding that sometimes these two things are merging together. The earlier attempts have been different. The related research literature seems to report no single type of intervention which is trying to focus on to as many ambitious goals as CG purports to reach. This explains why the means have-to-be also very different. All the goals have-to be almost necessary for reaching the FL.

It must be warned already now that reading comprehension requires so complex set of skills that we have to choose only the absolutely most necessary key aspects to be supported while other aspects are left to teachers. These ways of training, which the CG has-to included, are such that these can be open to be mediated to all.

The game has-to be first proven efficient empirically – as was made with the GraphoGame – before letting it to be sent to any ordinary use. The most appropriate dependent variable from which to judge the efficiency, is the observed better school achievements which results from how well the learners become able to learn knowledge from their schoolbooks. Thus, the comparison between ordinary instruction and that extended by the use of the CG, is the most appropriate way to observe the efficiency.

### 1.6. The goals and means of the support purported to be provided via ComprehensionGame

Let us first list the ten most important goals before explaining these. Each of the individual goal fulfills one aspect. Thus, the goal are:

1. to prepare children globally to learn the knowledge from their schoolbooks in a better way
2. to support children to comprehend and learn knowledge via reading in whatever language
3. to provide all the to-be mentioned supports to everyone who has the basic reading skill
4. to extend the learning of illiterate learners (after the use of the GraphoLearn Game) with ComprehensionGame to full literacy
5. to prepare learners to handle knowledge with appropriate consideration and critical thinking
6. to motivate teachers to learn to choose the essential information from the schoolbooks for her/his pupils
7. to confirm that the offered knowledge has become comprehended via follow-up assessment
8. to elevate/perfect in a long run the careful CG-users’ PISA results
9. to support also older than PISA-age learners to efficient learning of knowledge via reading
10. to offer means to good life to everyone to the extent it can be supported via instructing them confirmed knowledge by reading

The main goal of the CG is to train successfully comprehension of written knowledge to have, e.g., effective tools needed for learning in the school from the schoolbooks. The CG is meant to work – and start to be given into use preferably from ages (and up) of children who have just acquired the basic reading skill, i.e., the readiness to decoding written to spoken form.

The key mean the CG is using to reach the mentioned goals is to train the learner to become able to differentiate the most informative content from text which is less important in-order to help the learners to become able not only to comprehend but also to store the information for their future use. For reaching its goal CG had to be able to save working memory space of the user by helping s/he to identify key issues and concentrate on these thus making it possible to move the attended key information to the long-term memory. Overflow of the working memory cannot be learned if the learner is not able to identify efficiently the key content from the mass of text.

We also have the ambitious goal to develop this tool to help also, e.g., African illiterates. This can happen when African first and second graders have successfully learned the basic reading skill after which the learners need to take the described next step towards full literacy (FL) in their own language. They should be helped to be ready to take it also when learning to read the second language which happens typically starting from 3–5 grade. That this is possible is shown by our results from pilots made in rural Zambia where we have trained illiterate people to reach FL using GG and CG in sequence.

The learning environments meant to work globally should run in the cheapest phones which can be connected to Internet which starts to be available today also in rural Sub-Saharan Africa. Where it is not yet available, it will be available soon. This, at the same time, opens the ways to large variety of knowledge written in languages that the African children typically learn as their second language (English, French, Portugues, Spanish, and German) although some of the African languages, such as Swahili and Africans offer a lot of knowledge to their readers. But this is not the case of, e.g., Zambian learners to whom none of their hundreds of local variants of languages is offering sources of information whose content would be sufficient even for the most important needs of knowledge. E.g. the French version has been documented quite well (see, e.g., Lassault et al., 2022) as is the English version used for second language learning in India (Patel et al., 2018, 2022).

The next important skill children need today more and more is to train themselves to be able to read with critical reading, i.e., not believe everything which is available to them from written sources. Today critical reading skills are necessary for everyone, especially for children who do not have a self-evident readiness to approach information given to them with caution.

For these purposes the game should have appropriate contents and way to introduce those contents. The most natural content for children who have just learned to read is what the schoolbooks offer them. Therefore, the CG has to contain means to motivate teachers to implement the key contents from these books to the CG. This requires that the CG is sensitizing them to think carefully, which is the important key knowledge (for their students) in their schoolbooks, which children have to understand and store. In CG this information needs to be implemented in the CG with claims some of which (10%–30%) represent the typical misunderstanding children tend to have, to motivate children to carefully consider which is true and which is not – i.e., learn the critical reading skill. Children’s task is to judge each of the claims as being true or not. Only when all those judgements (chosen for a lesson) are correct the game is over successfully.

Typically, a game session contains a list of claims which covers one daily lesson. Reading the daily lessons from the schoolbook and playing the game summarizing the key information of the same text will slowly – after sufficient number of such learnings of lessons have been repeated – can make the learner able to apply the same ways of identifying the key information from the excerpt of the text to read whatever knowledge text. This elevates their readiness to master the ways of how to search the key information they had to store for comprehending the most important messages the text is telling them.

This all is based on the simple fact that not all words of the text are equal. Mostly the key information covers a relatively small portion of the length of the whole lesson. And teachers had to be able to choose the key content for pupils to learn comparably which often is the same as s/he does when choosing content for exams.

Such a use of the game is gradually motivating teachers and pupils to be critical when they approach written information because a central idea of the playing is based on learning from the critical judgements which they have-to do during the time they choose the text/play. Without being successful in this judging process, ie. making decisions needed of telling whether each of the claims is true or not, they will fail to have the game developed/played successfully and thus not mastering yet everything the CG is trying to guiding/instructing teachers/pupils.

To be able to have teachers to take the aforementioned duty into action, the teachers themselves had to feel that using this learning environment/application makes their work more effective and provides benefits which will save their time. Therefore, the game is programmed to follow the proceeding of the children’s learning via log/storage files, while the teachers can follow children’s achievements from CG comparably s/he can do by using exams. The game is built to be ending only after all the answers to the claims are given without any errors. This mostly requires a number of repetitions of answering to the claims which makes the storing the knowledge more reliable. And this naturally happens lesson-by-lesson which are given students daily. One game is one lesson. Thus, for each working day, when student has completed the daily lessons s/he has reached a full mastery of learning the content of the day.

The use of the CG for completing lessons after reading the lessons from the schoolbook slowly “conditions” pupils to be able to search the key content from any text given to them for learning. If this is continued for a long time before children reach 15 years of age, learners reading skill will approach full literacy (FL). Thus, they become ready to survive the PISAs reading tests, which OECD is organizing for literacy.

The CG had to be made working in all countries independently of language and schooling culture. Children should have someone, teacher or parent (of Community Schools in Africa), who is able to read and implement the content using the language that children have learned to read. In order to help the countries where the schooling is not yet good enough, the game offers a possibility to use appropriate knowledge content picked either directly from sources of the second language of the African pupils (mostly English from grade 4–6 depending on the country) or via their local language but picked via translation from whatever written source applicable to replace the local schoolbooks.

The very same property works also in such a way that instruction can be individualized for each learner according to her/his interest in learning something to keep the engagement sufficient. This may be important when we are teaching children who are otherwise reluctant to enjoy about school teaching. CG offers a possibility to choose whatever content according to the individual interest of the pupil. This may be an effective and working way to offer the knowledge which they would be happy to choose for earning a working place which is based on skills they are most interested in developing.

Guidelines which teachers need for understanding how the content can be implemented to the game are offering information about everything important and it is given also via the game itself to motivate teachers to experience how the game works.

The information given in the Comprehensiongame.info include also a lot of the content of this article and, e.g., summary description of the scientific basis of the work of CG. All is given not only in traditional way, in written form, but also in the very same game-format for the teachers to play than CG is using for supporting children to learn.

A lot of content is already available in the CG for helping also adults interested in the important knowledge to be learned efficiently. For example, in Finland we have implemented such content which is interesting to adults (fathers) to motivate them to play the game so as to have boys who need it most, to model what the fathers are doing. All this can be learned by skimming the ComprehensionGame.info pages where one can read also English knowledge content, for example, from climate change.

A very important goal is to make the tools user-friendly that both teachers and children accept starting to apply it both for implementing content from their schoolbooks to the game and to use such a “new” way to learn among children. Everything teachers had to do during a school day should be made as effective as possible that its use will last long enough for helping pupils to elevate their reading comprehension and use of visual language.

The final goal is to make CG engaging enough for those who need it even in the most literate country, Finland, so that they can reach FL. It has been shown by Progress in International Reading Literacy Studies (PIRS) that adult men often fail to be able to comprehend written language to the level they had to. Today it is boys whose PISA results have been falling which was observed into the three last times that literacy has been assessed. Most likely this happens because reading outside school is not among their interests anymore. Finnish girls are happy to follow today’s strategy that Finnish government has been trying to motivate children to read which is to invite children to collect “reading diplomas” which can be earned by reading certain number of books. It seems to work for them although also girls’ PISA results have been falling, too, which is not, however, alarming because the starting level of Finnish girls is still among the best in the world.

### 1.7. How CG differs from other existing intervention methods meant to support reading comprehension?

The CG learning environment is designed to reach/achieve all the set goals/aims needed for a person acquiring full literacy skills among children who have earlier learned the basic reading skills (which can be acquired using the GG). The majority of the previous interventions and studies have focused on training a FL in one specific writing system at a time, mainly in children who have had difficulties in acquiring basic reading skills. It also helps typical children who are not interested in leisure reading to acquire FL naturally. No one of the found intervention methods has been digital game-like training tool which can be used almost everywhere without needing any further programming. Thus the CG learning tool is able-to-be implemented also in post poor countries of the world. It can train people in the rural African areas as well, where books and practically all written material (but a few schoolbooks) is missing.

The CG environment can also motivate teachers to guide their students in a more effective way globally via a cheap phone and internet connections but also (but less optimal way) offline. Overall, there is no other intervention program which could cover attempts to reach as many goals as the CG does and it could be seriously considered to be used as a basic learning tool everywhere after it has been optimized using validation studies.

### 1.8. How we have started to study the support of FL using the CG

We have already had a relatively promising start. The present version has already shown to be effective enough to help rural Zambian illiterate people to learn important knowledge via the CG. The next group of active users has been teachers and children who are trained in a statewide network of schools (Valteri) working in Finland for helping children who have the greatest difficulties in learning. The teachers who have been most interested in it have wanted it to be more deeply gamified which hope has now been followed and first gamified versions are now under use especially among some second-grade boys.

Because CG allows the development of individualized games taking into account the readiness of the player to benefit from the content, it has brought happiness to the teachers of these schools. CG makes their life much easier because they can build databases of content to a big variety of different learners, and they do not always need to start always from the beginning when teaching a new learner who has a certain kind of difficulties in his/her learning.

Everyone who wishes to join working for the mentioned goal is invited to contact me if the game when tried from comprehensiongame.info looks interesting. I hope we can keep it open to be used for research until the time you are reading this. For learning about that, please, write to me to heikki.lyytinen@comprehensiongame.com.

### 1.9. The plans how to validate the ComprehensionGame

The validation research of the CG is now running in rural Zambia and will soon cover many studies at different grade levels in Finnish schools. The possibility to individualize instruction using CG is easy to fit to the individual’s readiness to learn, when support is needed, it will be tried in many ways in the near future in Finland.

One plan is to propose it to be used concurrently city-wide at different grade levels using experimental designs which allow collecting convincing evidence about how well the use of CG can affect on school achievements. Also, implementing CG to leisure time activities for 2 and 3 grade children, who spend their afternoon times in school services, is running in a form where its effects on school learning and interest in using it is observed. The final and most ambitious plan is to motivate it into free use among all Finnish second graders during the autumn semester of 2023. The country-wise experimental studies with big random samples will be available for everyone who has sufficient research experience or who accepts my supervision.

The final goal is that the validated version will help as many as possible who have the basic reading skill in the world, to reach full literacy. Then an ideal way to have it made available to people in each country would be some kind of state procurement, a kind of financing the opportunity similar to what was applied in Finland when the Ekapeli (GraphoGame outside Finland) was developed more than 10 years ago. Now Ekapeli’s availability as a free digital learning environment for learning the basic reading skill among Finnish children has been kept free to all Finnish children. A comparable procedure would be best in terms of help all in need in any country who wanted to organize the use of such an opportunity to its learners.

The running of the wanted efficiency studies is easy because the program is automatically logging all the research data for which the organizer of the study has got appropriate permissions of use. Also, the initial analyses will be run almost entirely online to-be-followed by the researchers who typically make their studies in departments such as psychology, education, teacher education and can be organized by using whatever language.

As soon we have optimized the game to be good enough comparative studies will be planned to be initiated in Europe via the cooperation of the second author with the help of the first author. The first author is willing to supervise research of CG elsewhere, including Africa. We are searching interested colleagues to start considering comparable studies in their own countries to which the CG is given for free for research purposes.

## 2. Discussion

We have described above our plans which are partly already realized – such as providing our GraphoLearn technology be used for supporting millions of children in tens of countries under the label GraphoGame – and those which are in piloting phase – such as the ComprehensionGame. The early results reveal that we have been able to support with the mentioned two digital, game-like learning environments (CL and then CG) also illiterate Zambian rural people to learn knowledge via reading. The to-be-made operations are the empirical documentation that all the goals taken for the ComprehensionGame to reach, can be empirically shown to work.

In Zambia and other Sub-Saharan African countries the main reason why children are not becoming literacy is the lack of interesting reading material. This is not easy to correct especially in rural Africa. This is why we are now developing a training environment which is based an further development of the use of so called statistical learning principle (see Arciuli, 2018), which could be used already before school age. The time to write more about this is after our empirical testing have been completed in Africa.

Related research operations of the CG have been started in Zambia and in Finland where children are ready to start (i.e., are fluent readers) from the second graders but helpful also during later grades. This all is possible because the game is age and language independent which means that also that the FL of the second language such as English can be easily implemented in the CG.

It is important to note that those who are also in need of support can be found also from typical learners especially among boys who do not read outside school. This is why in many countries including Finland the PISA results are falling among boys. They have-to-be motivated to us CG to approach written language so that they can learn to read efficiently with understanding at least the schoolbooks.

### 2.1. Limitations

A main limitation affecting our work seems to be the difficulty of collecting the needed funding which of course defines the speed of proceeding in reaching our ambitious goals. And for that we have to have sufficient initial evidence documenting the efficiency of this almost unbelievable approach. Happily, the way we collected the needed evidence for the GraphoGame seems to work also for documenting the efficiency of the ComprehensionGame. Students running their doctoral studies have shown comparable interest in helping us in running the needed studies. And this will not need too much funding because I am open to supervise without payment if someone wants to find a local supervisor who accepts me working as the main supervisor for the student to run her/his PhD studies and defend these in one’s own university. Where funding is needed is, however, in poor countries where only few have a phone or tablet which can be connected to internet. Good advises are needed for solving this problem.

## Author contributions

HL designed the intervention tool, contributed to African and Finnish operations for validation and interpreted the results of the intervention tool. HL and NL drafted and revised the review critically for important intellectual content and prepared and edited the draft and the final manuscript. All authors contributed to the article and approved the submitted version.

## Conflict of interest

The authors declare that the research was conducted in the absence of any commercial or financial relationships that could be construed as a potential conflict of interest.

## Publisher’s note

All claims expressed in this article are solely those of the authors and do not necessarily represent those of their affiliated organizations, or those of the publisher, the editors and the reviewers. Any product that may be evaluated in this article, or claim that may be made by its manufacturer, is not guaranteed or endorsed by the publisher.
